# Bone Turnover in Wild Type and Pleiotrophin-Transgenic Mice Housed for Three Months in the International Space Station (ISS)

**DOI:** 10.1371/journal.pone.0033179

**Published:** 2012-03-15

**Authors:** Sara Tavella, Alessandra Ruggiu, Alessandra Giuliani, Francesco Brun, Barbara Canciani, Adrian Manescu, Katia Marozzi, Michele Cilli, Delfina Costa, Yi Liu, Federica Piccardi, Roberta Tasso, Giuliana Tromba, Franco Rustichelli, Ranieri Cancedda

**Affiliations:** 1 Dipartimento di Oncologia, Biologia e Genetica, Università degli Studi di Genova, Genova, Italy; 2 Istituto Nazionale per la Ricerca sul Cancro, Genova, Italy; 3 Dipartimento di Scienze Cliniche Specialistiche ed Odontostomatologiche, Università Politecnica delle Marche, and Consorzio Nazionale Interuniversitario per le Scienze fisiche della Materia, Ancona Unit, Ancona, Italy; 4 Department of Industrial Engineering and Information Technology, University of Trieste, Trieste, Italy; 5 Sincrotrone Trieste S.C.p.A., Basovizza (Trieste), Italy; 6 Istituto Nazionale Biostrutture e Biosistemi, Roma, Italy; Ohio State University, United States of America

## Abstract

Bone is a complex dynamic tissue undergoing a continuous remodeling process. Gravity is a physical force playing a role in the remodeling and contributing to the maintenance of bone integrity. This article reports an investigation on the alterations of the bone microarchitecture that occurred in wild type (Wt) and pleiotrophin-transgenic (PTN-Tg) mice exposed to a near-zero gravity on the International Space Station (ISS) during the Mice Drawer System (MDS) mission, to date, the longest mice permanence (91 days) in space. The transgenic mouse strain over-expressing pleiotrophin (PTN) in bone was selected because of the PTN positive effects on bone turnover. Wt and PTN-Tg control animals were maintained on Earth either in a MDS payload or in a standard vivarium cage. This study revealed a bone loss during spaceflight in the weight-bearing bones of both strains. For both Tg and Wt a decrease of the trabecular number as well as an increase of the mean trabecular separation was observed after flight, whereas trabecular thickness did not show any significant change. Non weight-bearing bones were not affected. The PTN-Tg mice exposed to normal gravity presented a poorer trabecular organization than Wt mice, but interestingly, the expression of the PTN transgene during the flight resulted in some protection against microgravity’s negative effects. Moreover, osteocytes of the Wt mice, but not of Tg mice, acquired a round shape, thus showing for the first time osteocyte space-related morphological alterations *in vivo*. The analysis of specific bone formation and resorption marker expression suggested that the microgravity-induced bone loss was due to both an increased bone resorption and a decreased bone deposition. Apparently, the PTN transgene protection was the result of a higher osteoblast activity in the flight mice.

## Introduction

Bone is a complex dynamic tissue undergoing a continuous rebuilding-destroying process (remodeling) throughout a lifetime in order to adjust to mechanical demands, to prevent accumulation of fatigue damage, to repair micro-fractures, to ensure the viability of the osteocytes and to participate in calcium homeostasis. The remodeling process is characterized by a rapid resorption and a slower formation phase. Two different cell types play a major role in the process: osteoblasts, the bone-forming cells, and osteoclasts, the bone resorbing cells. Osteoblasts are differentiated cells derived from lining cells, the immediate precursors residing on the bone surface, that regulate the deposition of the bone matrix molecules, including type I collagen and a variety of other non-collagenous proteins. Osteoblasts become osteocytes as soon as a mineralized matrix surrounds them. Instead, osteoclasts, responsible for the mineralized bone matrix resorption, are multinucleated giant cells formed by the fusion of mononuclear progenitors of the monocyte/macrophage lineage.

Bone cells act as one extraordinary orchestra in order to increase or decrease skeletal mass on the basis of its degree of utilization. The skeleton main role is to sustain locomotion that has to be performed counteracting the Earth gravity. When the skeleton has not to stand against gravity, as it happens during spaceflights, exercise and movements are reduced leading to a decrease of whole bone mass and density and making bones brittle [1]. Early in the space program, studies performed on Gemini and Apollo mission crew showed a severe bone demineralization associated with an increase of calcium and phosphorus excretion in the astronauts. Skylab missions allowed additional studies that highlighted how the astronaut serum levels of bone formation markers decreased whereas urinary markers of bone loss increased over 30% [2]. Studies on cosmonauts that were aboard the Russian MIR space station provided irrefutable data derived from longer missions. Indeed, Russian crewmembers experienced a significant bone loss in the tibia already within 1 month, which continued throughout the flight period up to 6 months [3]. This bone loss was observed in both trabecular and cortical bone compartments. Interestingly, spaceflights did not alter bone mass in the non weight-bearing radius. Post-flight follow-up investigations revealed that a recovery time in normal gravity of the same length of the flight duration was insufficient to restore the bone mineral density at a level corresponding to the pre-flight status [4]. Consistently with results from humans, also the bone loss observed in rats after flight was not recovered by re-ambulation in normal gravity for a time corresponding to the flight duration [4]. Most recently Dual-emission X-ray Absorptiometry (DXA) measurements performed on fourteen crewmembers that were aboard the International Space Station (ISS) for 4–6 months revealed bone loss at an average rate of 0.8% and 1.5% per space/month in the lumbar spine and in the femur, respectively. The determination of the bone loss rates provided also further evidence that space permanence affected the trabecular bone more severely than the cortical bone compartment [1], [3].

Bed rest is the most reliable on-Earth system to unload a human skeleton. Some studies showed that bed rest (up to one year) caused in volunteer subjects an uncoupling between bone formation and resorption characterized by a very rapid bone loss [5]–[7]. In these volunteers, over a period of 4–8 months, the monthly calcium loss and the urine phosphorus excretion was approximately 0.5% [8], [9]. The calcium and phosphorus mobilization from bone could possibly explain the observed concomitant changes in the serum levels of major calcium-regulating hormones as seen also in astronauts after spaceflights [9]–[12]. Interestingly, during the bed rest an increase of about 3% in the bone density of the skull was observed. This finding may imply redistribution rather than a net loss of the bone mineral where head bones, that are not weight-bearing, may act as an *in vivo* storage site for the mineral released from other skeletal sites. This density increase could be the consequence of an increased hydrostatic pressure in the head respect to the leg, as it occurs also during a spaceflight due to a cephalic fluid shift [6]. In a 360-day bed rest study, etidronate, a drug blocking osteoclast maturation, was a countermeasure more effective than exercise in preventing the loss of bone mineral density in the femoral neck [13], [14]. These data suggest that, as in the case of spaceflights, the primary response of the skeleton to prolonged bed rest is an increase in bone resorption by osteoclasts with a parallel release of calcium and phosphorus from bone tissue to the circulation. Except for the osteoclast tartrate resistant alkaline phosphatase (TRAP), the altered expression of biochemical markers of bone metabolism rapidly returned to normal during the recovery period [9], [15]–[17]. Because of the paucity of the involved subjects and the limits in the opportunity to study microgravity effects on human bone, several studies related to bone loss in space have been performed taking advantage of rat or mouse models. Most of the data from these animal studies were in agreement with those obtained from human studies, nonetheless investigators have to take into account that results have to be interpreted considering the specific experimental conditions, such as animal age, sex, body weight, pregnancy and variable delay in post-flight sample collection (i.e. sacrifice of the animals), that could affect the final results. Early studies conducted in Wistar rats during a 19.5-day spaceflight indicated a reduction in periosteal bone formation and in the longitudinal bone growth evidenced by the appearance of an extensive arrest line in the cortical bone periosteum and a decrease in the primary spongiosa width [18]. Interestingly, the bone formation rate was not uniformly depressed in the tibia cross-section and was less severe at the level of the anterior tibia crest, where muscles are inserted [19]. This evidence suggests that the deleterious effects of mechanical unloading in microgravity can be partially avoided by muscular contractions [20]. A more recent study showed that bone resorption rate in rats exposed to 20-day spaceflight remained comparable to the one of ground controls [21] whereas several other studies confirmed a reduced presence and activity of osteoblasts in the flight animals and demonstrated the existence of site-specific changes in bone resorption [22]–[25].

Spaceflights lead to a decrease in osteoblast number and activity [22], [26], [27], most likely as result of an altered differentiation of osteoblast precursors. In rats exposed for 1-week to the space environment during the Spacelab 3 mission, tibial osteoblasts had a smaller cytoplasmic area, possibly consequence of the reduced collagen secretion [22]. In the same animal model, most studies showed that osteoclast number and activity remained unchanged in spaceflight animals. An increase in the osteoclast population was only observed in different bone sites of pregnant rats after a space permanence of less than one week [28]. A 14-day period in space provoked a decrease in gene expression of bone matrix proteins, including osteocalcin, osteonectin, and type I collagen in Sprague Dawley rat weight-bearing bones together with a reduction of osteoid surface and trabecular bone volume [29]. When the differentiation of the cells of the osteogenic lineage to more mature forms was inhibited, the expression level of two characteristic bone formation markers, such as alkaline phosphatase and osteocalcin, changed in an opposite direction [30].

Studies on rats exposed to microgravity revealed that the alkaline phosphatase mRNA levels in serum and bones increased whereas osteocalcin mRNA levels decreased after flight. [31], [32].

In this paper we report investigations on skeleton alterations occurred in mice exposed to a near-zero gravity in the International Space Station (ISS) for 3 months during the Mice Drawer System (MDS) mission (Shuttle Discovery Flight 17A/STS-128 on August 28^th^, 2009. MDS re-entried on November 27^th^, 2009 with Shuttle Atlantis Flight ULF3/STS-129 after 91 days), the longest permanence (91 days) of mice in space. For the MDS experiment, in addition to Wt mice, transgenic mice over-expressing pleiotrophin (PTN) under the control of the human bone specific osteocalcin promoter (PTN-Tg) were selected. Pleiotrophin (PTN) is an extracellular matrix-associated growth/differentiation factor widely expressed in embryonic development [33], [34]. However in postnatal life PTN is found mainly in bone and brain [34]–[36]. Together with midkine [37], [38] and the chicken protein retinoic acid-induced heparin-binding protein (RIHB) [39], PTN defines a new family of secreted heparin-binding growth factors structurally unrelated to other growth factor families. PTN has different functions, ranging from stimulating neurogenesis, cell proliferation, and chemotaxis to tumor angiogenesis [35], [37]–[40].

PTN is considered an osteotrophic agent because mice overexpressing the human *ptn* gene appear more protected than Wt with regard to the mineral loss of bone occurring during aging [41]. Moreover PTN-transgenic mice compensate for the bone loss observed after ovariectomy [42].

At cellular level, PTN was chemotactic to a variety of osteoblastic cell line [43] and osteoprogenitors from human bone marrow [44], suggesting that pleiotrophin might play a role during bone remodeling by attracting osteogenic cells to new bone formation sites. Indeed, PTN was expressed in sites of new bone formation [41], [43]. PTN was synthesized by osteoblasts at an early stage of development and enhanced the osteogenic differentiation of mouse and human bone marrow derived cells [41], [44].

Recently Imai et al. reported a significant decrease in the bone mass of the weight-bearing bone in PTN−/− mice compared to wild type controls [45]. These authors extended their analysis to prove an association of PTN to bone formation after mechanical loading. KO mice did not respond normally to bone disuse suggesting a role of PTN in mediating the adaptation of bone to environmental cues.

Absence of gravity and mechanical unloading are the major cause of bone loss in space. Indeed, several papers associated PTN with bone formation and mechanical loading [46]–[48].

We selected PTN-Tg mice to investigate whether these mice were protected from space related osteoporosis and whether the PTN over-expression could be considered a countermeasure for the bone loss observed in microgravity.

## Results

### Flight samples and ground controls

One of the major goals of the MDS experiment was to investigate bone alterations in 3 Wt and 3 PTN-Tg male mice (2-months old at the time of launch) after 3-month permanence in the ISS, the longest permanence of mice in space. Unfortunately only three mice (Wt2, PTN-Tg1 and PTN-Tg2) returned to Earth alive at the end of the mission. Three mice died during the flight: Wt3 after 16 days; PTN-Tg3 after 24 days; Wt1 after 44 days. Astronauts immediately froze the dead mice in order to store them for subsequent computed microtomography (μ*CT*) analysis on their bones. The necropsy revealed that mouse Wt3 had a major spinal cord lesion possibly occurred during the shuttle lift off. An analysis performed on feces present in the cage of the PTN-Tg3 mouse suggested that the animal could have developed a liver pathology. The post–landing checking performed on the mice cages revealed that Wt1 mouse died in consequence of a failure of the food cassette system. (More datails are given in the companion article of this collection “The Mice Drawer System–MDS–Experiment and the Space Endurance Record-Breaking Mice” by Cancedda et al.).

**Figure 1:**
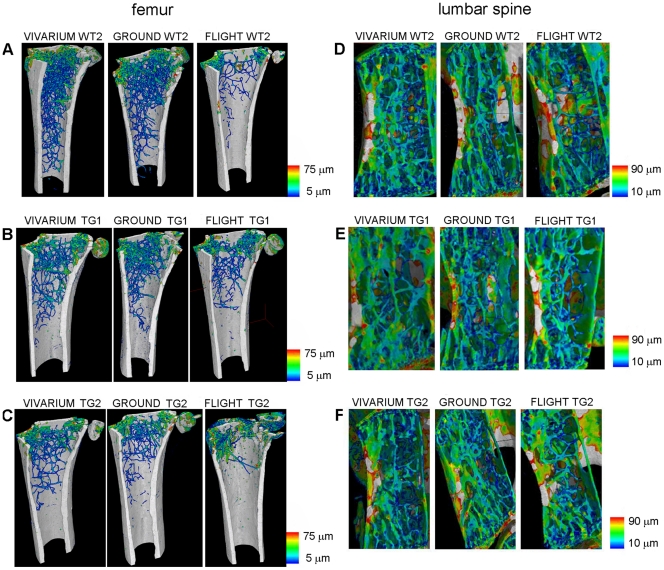
Bone trabecular thickness distribution. Wt2 (A) PTN-Tg1 (B) PTN-Tg2 (C) color map of bone trabecular thickness distribution in the femur of a representative vivarium, the ground and the flight mice. Wt2 (D) PTN-Tg1 (E) PTN-Tg2 (F) color map of bone trabecular thickness distribution in the seventh lumbar vertebra of representative vivarium, the ground and the flight mice.

To check MDS habitat impact, other than microgravity, on flight mice, a ground replica of the flight experiment was conducted housing six mice in the MDS spare model for three months. During the ground replica of the experiment, three mice (Wt1, Wt3 and PTN-Tg3) were sacrificed exactly at the same experiment day of the death of the mice on the ISS. Moreover three Wt and three PTN-Tg mice of the same age maintained in standard cages and conditions (vivarium), were considered as further controls. Values derived from the vivarium samples were averaged in all types of analysis, thus considering only one value per experiment data.

**Figure 2:**
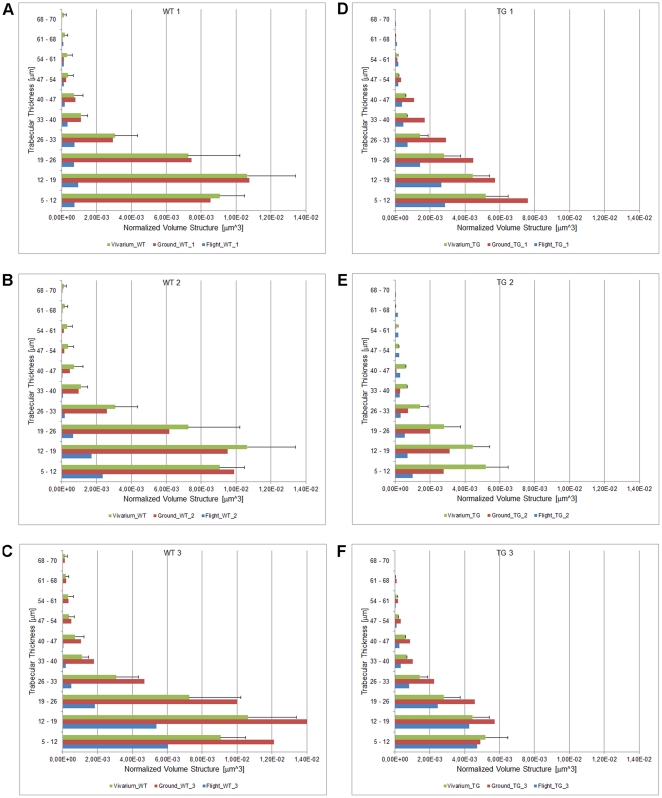
Quantification of trabecular thickness distribution in femurs. Wt (A-C) and PTN-Tg (D-F) flight experiment, ground and vivarium control mice. The value reported for the vivarium condition is the average of the three animals.

### Computed microtomography (μCT) analysis on weight-bearing bones

Since different bones respond to microgravity in a different way depending on their intrinsic characteristics and the length of the exposure, we analyzed both weight-bearing (femur and lumbar spine) and non weight-bearing bones (calvaria) of all animals. Computed microtomography (*μ*CT) image analysis was performed in order to characterize the three-dimensional bone microarchitecture. Among the femurs of the vivarium mice examined, Wt mice showed a mean trabecular number (Tb.N) higher than PTN-Tg mice and a narrower mean trabecular separation (Tb.Sp) (statistical t-test with p < 0.05, Table S1, S2) confirming some preliminary unpublished observations of ours. Although an effective statistical analysis was not performed due to the reduced number of samples, in all femurs of both Wt and PTN-Tg mice exposed to microgravity a significant decrease in the bone volume to total volume ratio (BV/TV) values as well as in the connectivity index (β) (data not shown) with respect to the femurs of ground and vivarium control mice was observed. The computed trabecular number (Tb.N) value confirmed the tendency observed for the BV/TV parameter, resulting decreased in the flight samples compared to ground and vivarium controls. Instead, no significant changes in the mean trabecular thickness (Tb.Th) were observed in the animal femurs regardless of the three different experimental conditions. Consequently, also the specific surface of the trabecular structure (BS/BV) was a relatively stable parameter: the determined values were comparable in both Wt and PTN-Tg and did not dramatically increase after flight. Coherently with the previously considered parameters, the Tb.Sp increased in all the samples after flight. Moreover, the color map of bone trabecular thickness distribution in the femur of vivarium, ground and flight mice confirmed a significant decrease of the Tb.N values in both Wt and PTN-Tg mice that experienced microgravity conditioning (Fig. 1A-C). Also by this type of analysis, after the three months exposure to microgravity, in the two PTN-Tg mice the trabeculae appeared in percentage less reduced than in the Wt2. To investigate more deeply trabecular changes, the trabecular thickness distribution vs. the trabecular volume normalized to the total sample volume was also assessed. Histograms of the distribution of the trabecular thickness in all mice are reported in Figure 2. The trabecular thickness distribution of the Wt3 mouse, which died on the 16^th^ mission day, was already altered by the microgravity exposure. The alteration of the trabecular distribution in the Wt1 mouse that died after 44 days was even more severely affected and comparable to the one observed in the Wt2 mouse that survived to the flight. On the contrary, the PTN-Tg3 that died after 24 days presented a trabecular thickness distribution similar to the one of the PTN-Tg ground and vivarium controls.

**Figure 3:**
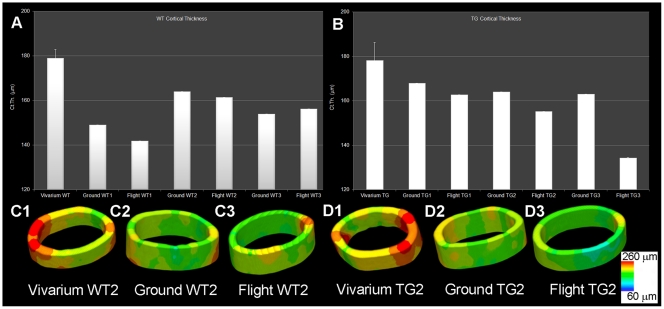
Quantification of cortical thickness distribution in femurs. Wt (A) and PTN-Tg (B) flight mice, ground and vivarium control mice. The value reported for the vivarium condition is the average of the three animals. In the bottom panels cortical thickness color maps of representative 3D reconstructions of the same cortical region in Wt2 vivarium, ground and flight (C1-3) and in PTN-Tg2 vivarium, ground and flight (D1-3), are shown.

**Figure 4:**
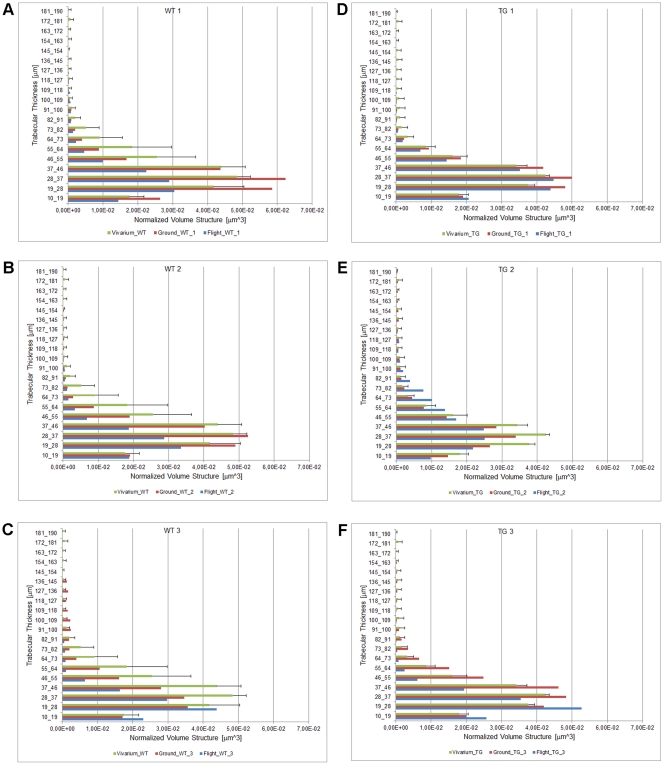
Quantification of trabecular thickness distribution in vertebral columns. Wt (A-C) and PTN-Tg (D-F) flight, ground and vivarium control mice. The value reported for the vivarium condition is the average of the three animals.

It has been reported that microgravity exposure affects not only trabecular but also cortical bone. The *μ*CT analysis revealed also a reduced cortical thickness in both the flight Wt and PTN-Tg mice compared to the corresponding vivarium controls and to a lesser extent to the ground controls, as it is also evident by the 3D reconstruction (Fig. 3) made adopting a thickness color map similar to the one used for Figure 1. Interestingly, the Wt and the two PTN-Tg mice that returned to Earth alive were affected no more than the mice that died during the flight. At variance with observations made in femurs, no significant differences were noticed for all the investigated morphometric parameters when comparing lumbar spines of vivarium Wt and PTN-Tg mice (statistical t-test with p>0.05, Table S3, S4). Instead, coherently with the femur observation, an important reduction of the BV/TV ratio and of the connectivity index in the Wt lumbar spines was observed after the flight. Moreover, after the microgravity conditioning, there were evidences of alteration also in the mean number (Tb.N) and in the separation (Tb.Sp) in both Wt and in PTN-Tg mice. In particular, these were more pronounced in Wt mice suggesting a possible protection of the transgene expression against flight bone loss. The color maps and the histograms of the distribution of trabecular thickness in lumbar spines are reported in Figure 1 D-F and Figure 4, respectively.

All acronyms reported in this article are explained in Table S5.

**Figure 5:**
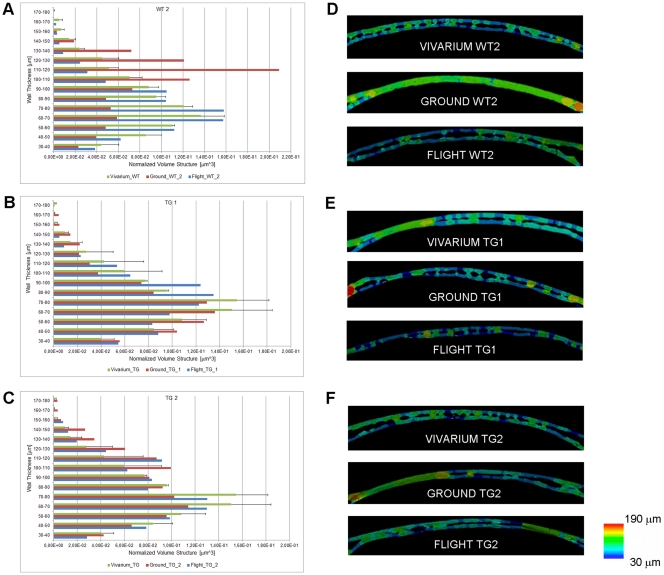
Non-weight-bearing bones analysis. On the left: quantification of bone thickness distribution in the calvaria of Wt2 (A) PTN-Tg1 (B) PTN-Tg2 (C) obtained from the *μ*CT analysis. On the right: parietal bone color maps of the Wt2 (D) PTN-Tg1 (E) and PTN-Tg2 (F) of flight, ground and a representative bone of the vivarium control.

### Computed microtomography (μCT) analysis on non weight-bearing bones

It is widely accepted that non weight-bearing bones are very slightly or not affected by microgravity exposure. A *μ*CT analysis limited to the bone thickness assessment was performed on the calvaria and, more precisely, on the parietal bones of Wt2, PTN-Tg1 and PTN-Tg2 (Fig. 5). No differences were observed in the calvaria thickness between Wt and PTN-Tg mice of the vivarium. The two transgenic PTN-Tg1 and PTN-Tg2 mice presented a thickness comparable to both ground and vivarium groups and showed no evident alterations in the calvaria thickness after the three months in space (Fig. 5B, C). The flight Wt2 had a parietal thickness comparable to the averaged one of the vivarium controls, however presented a thinner parietal bone than the corresponding ground Wt2 mouse (Fig. 5A). Nonetheless, the peculiar shape of the calvaria of the ground Wt2 mouse–lacking the marrow space between the two layers of the parietal bone (Fig. 5D)–suggested the occurrence of a very unique morphology or even a malformation in the bone of this animal (Fig. 5A). A color map representing the calvaria thickness, and confirming the made observations, is shown in Figure 5D-F.

**Figure 6:**
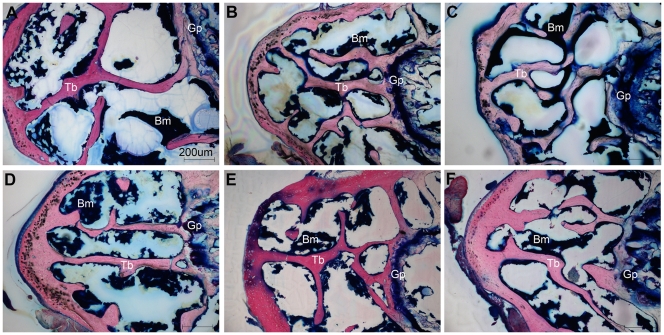
Histology on femurs on flight samples. Stevenel’s/Van Gieson staining was performed on the epiphyseal region of the same *μ*CT analyzed femurs. Flight Wt2 (A) ground Wt2 (B) vivarium Wt2 (C) flight PTN-Tg2 (D) ground PTN-Tg2 (E) and vivarium PTN-Tg2 (F) mice trabecular femur bone (B). Bm = bone marrow, Tb = trabecular bone, Gp = growth plate.

**Figure 7:**
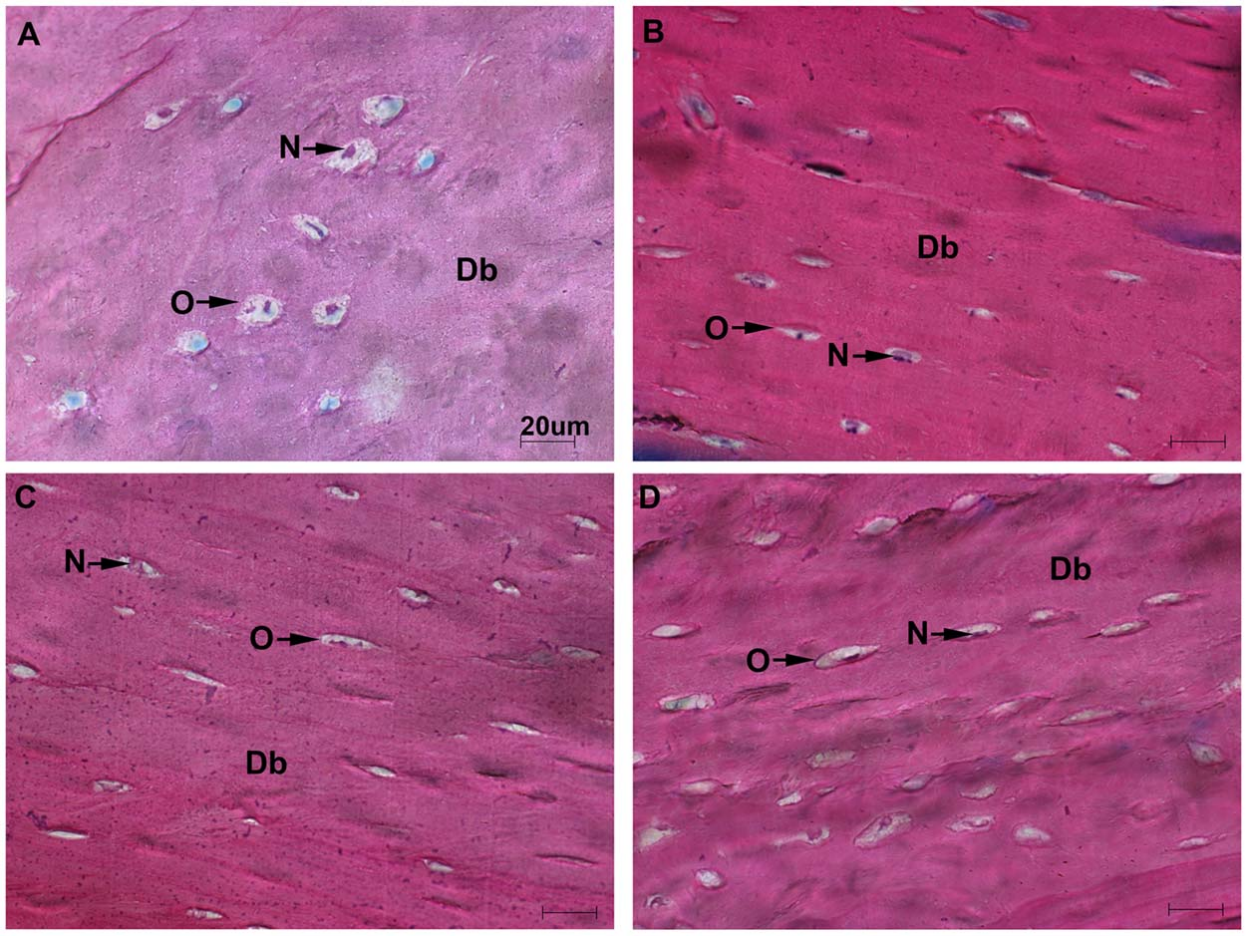
Osteocytes morphology. Stevenel’s/Van Gieson staining was performed on the diaphysial region of the same *μ*CT analyzed femurs. Flight Wt2 (A) ground Wt2 (B) flight PTN-Tg2 (C) and ground PTN-Tg2 (D) cortical bone, magnification 60x. Db = diaphyseal bone, O = osteocytes, N = nucleus.

### Microgravity sensing by mature osteocytes

On the same samples previously analyzed by *μ*CT we subsequently performed histology. After staining, the tissue slices from Wt and PTN-Tg bones were scored under the microscope and compared for tissue morphology and organization. Although, due to the previous laser treatment, the quality of the obtained images was reduced, the histology analysis clearly showed a reduction in the trabecular compartment of flight bones thus confirming the *μ*CT findings (Fig. 6). Moreover the histological analysis also revealed some differences between osteocytes of flight and ground bone diaphysis. Osteocytes are mature stellate cells surrounded by mineralized bone matrix that, acting as mechanosensors, are responsible for maintaining bone integrity. Wt2 flight mouse bone osteocytes appeared bigger, rounded in shape and surrounded by a larger lacuna compared to PTN-Tg flight mice osteocytes (Fig. 7, Fig. S1). To support the visual observations, we measured osteocyte dimensions in random fields of histology slides from all the samples. Statistically significant differences were found between flight Wt (7.3 µm±1.8, n = 61) and flight PTN-Tg (5.6 µm±1.3, n = 63) as well as between ground Wt (5.8 µm±1.7, n = 34) and flight Wt samples (Student’s t test, p<0.0001 and p = 0.0001 respectively). No statistically significant differences were found between flight PTN-Tg and ground PTN-Tg (average 5.8 µm±1.9, n = 72) samples as well as between ground Wt and ground PTN-Tg samples (Student’s t test, p<0.5064 and p<0.8561 respectively).

**Figure 8:**
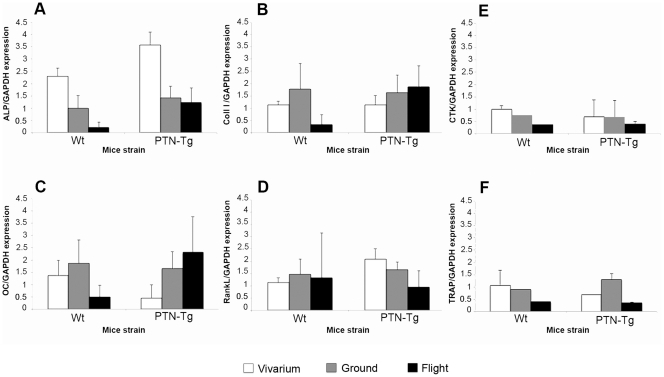
Molecular evaluation of typical markers for bone formation and resorption. Real time PCR on cDNA samples from RNA extracted from flushed humerus and tibia for typical bone formation and resorption markers such as ALP, Coll I, OC and RankL. TRAP and CTK were evaluated by Real Time PCR on RNA extracted from the bone marrow of the flushed humerus and tibiae. RNA samples were from flushed bone and bone marrow samples derived from the Wt2, PTN-Tg1 and PTN-Tg2 survived mice.

**Figure 9:**
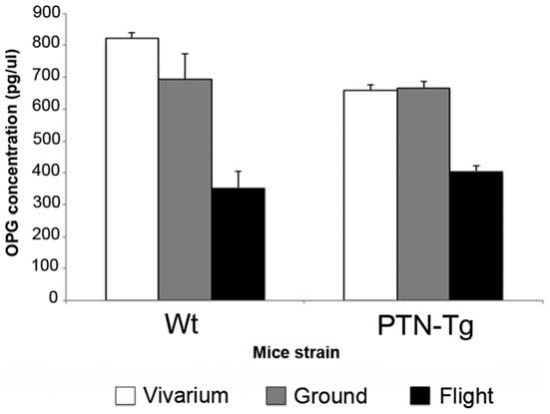
Serum OPG level analysis. Luminex^®^ assay was performed on serum samples of flight and ground Wt2 and an average of flight and ground PTN-Tg1, PTN-Tg2 as well as an average of Wt1, Wt2, Wt3 and of PTN-Tg1, PTN-Tg2 and PTN-Tg3 vivarium mice in order to measure serum OPG level.

### Molecular analysis of bone formation and resorption markers

RNA was extracted from combined tibia and humerus bones and separately from bone marrow of the flight, ground and vivarium mice. The expression of typical bone formation markers was analyzed by Real Time-PCR (Fig. 8). A decreased level of mRNA for alkaline phosphatase (ALP), collagen type I (Coll I) and osteocalcin (OC) was observed in the flight Wt2 mouse when compared to vivarium and ground samples and to mRNA level in a pool of the RNA extracted from flight PTN-Tg1 and PTN-Tg2 flushed marrows. The observed tendency of PTN-Tg mice to maintain a higher expression of the bone formation markers was in line with the hypothesis, based on the *μ*CT results, that the PTN transgene had a protective effect against the bone loss consequent to the three months microgravity exposure. No remarkable differences were measured in all RNA samples with regard to the expression of an osteoblast gene, such as Rank ligand (RankL), that plays a role in the induction of osteoclast maturation. Moreover no differences between Wt and PTN-Tg were observed with regard to the expression of the osteoclast markers tartrate-resistant acid phosphatase (TRAP) and cathepsin K (CTK) except for a slight decrease in the CTK mRNA level in the sample from the flight Wt. The variability in the different mRNA levels observed in the samples made difficult to interpret these results and to draw definitive conclusions. However, a Luminex® analysis revealed that in the blood serum of both flight Wt and flight PTN-Tg mice the protein amount of the RankL antagonist, osteoprotegerin (OPG), was significantly reduced suggesting an involvement of bone resorption in the observed bone loss in both Wt and PTN-Tg strains (Fig. 9).

## Discussion

Genetic background and several pathologies can modify bone physiology inducing either osteopetrosis, characterized by a higher bone mass, or osteoporosis, a condition linked to decreased bone integrity and strength. Bone mechanical loading, such as during physical exercise, leads to an enhanced strength, while an unloading condition, such as during bed rest, depletes the bone matrix rendering the bone more fragile. Gravitational force significantly affects bone remodeling. Past space missions evidenced the importance of gravitational force on the maintenance of bone integrity. On Earth, bones and muscles of the legs and lower body are normally required to maintain an upright posture and therefore they have to work against the force imparted by gravity on the body mass. Exposure to near-zero gravity leads to a progressive bone mass loss and it has been proposed as an accelerated osteoporosis model. Prior to our work, different animal models were used to study microgravity effects on skeleton. In particular, wild type rats were sent to space on the occasion of few, mainly Russian, missions. At variance with our MDS experiment, in all previous cases the animal space permanence was only for a limited time period.

We had the possibility to investigate skeleton alterations occurring in Wt and PTN-Tg mice exposed to a near-zero gravity in the ISS for 3 months, the longest permanence in space of mice. The use of mice for scientific research aims offers several advantages. Mice can easily be housed in cages smaller than those used for bigger animals such as rats; moreover, genetic modifications can make mice a reliable model, for example, of skeletal alterations since the murine skeleton shows molecular and morphological characteristics that are very similar to the human ones. The transgenic mice we selected for the MDS experiment overexpressed PTN in the bones. This particular transgene was chosen because it is a growth factor that exerts a positive effect on bone formation [49] and because of its association with bone formation after mechanical loading [45]. Our guess was that PTN could also protect against the bone loss due to the reduced gravity. Due to the hardware and mission constraints, we could fly only 6 mice (3 Wt and 3 PTN-transgenic). Unfortunately, as mentioned before, three mice died during the mission. Nonetheless, astronauts froze them immediately after death, and on return on Earth, we succeeded in performing histomorphometric μCT measurements on the bones of these dead mice. Undoubtedly, the MDS payload presented a technical limitation regarding the number of animals housed and unfortunately only three mice survived to the MDS mission creating a critical situation about the possibility to perform a reliable statistical analysis. However, the MDS experiment represented a unique opportunity to perform an experiment designed to study the effect of a long-term exposure to a microgravity environment on bone metabolism and turnover and to collect data that could guide the planning of future similar experiments. Eventually these results could also favor the development of countermeasures for astronauts exposed to the risk of a serious bone loss in future long term space missions to the Moon or Mars.

Bone architecture reflects bone functionality and behavior. Here we showed that Wt mice maintained in standard vivarium conditions for 5 months (experiment controls) presented a more complex trabecular organization in terms of mean trabecular number than PTN-Tg mice of the same age and maintained in the same vivarium conditions. This finding was in agreement with previous published data reporting a greater bone mass in Wt mice than in PTN-Tg mice only from the 25^th^ week of age [41]. In flight mice of both strains (Wt and PTN-Tg), mean trabecular number was decreased. Trabecular thickness distribution plots suggest that, most probably, thicker trabeculae became thinner whereas initially thinner trabeculae (at the limit of the 9–10 µm in linear size) were no more detectable because they became thinner than the experimental voxel size. The fact that during flight Wt type mice tended to lose more trabeculae than PTN-Tg mice, thus reducing the differences between the two mouse strains, suggests that the expression of the PTN transgene exerts some protection on the skeleton against the bone loss consequent to the microgravity exposure. This was in agreement with the observations made on ground by Imai et al. which reported a clear osteopenic phenotype in the adult PTN−/− mice [45]. This transgene “protective role” against the bone structure deterioration was not observed for cortical bone. In fact, the *μ*CT analysis evidenced a comparable reduced cortical thickness after the flight, in both Wt and in PTN-Tg mice. Indeed the MDS habitat *per se* caused a reduced cortical bone thickness, most probably, as result of the reduced movement allowed in the payload compared to vivarium cages.

Tare et al. reported thicker calvaria in neonatal PTN-Tg mice compared to Wt mice [41]. We did not observe a difference between the width of the calvaria in Wt and PTN-Tg mice probably because of the different age and/or genetic background (C57BlJ10 versus BDF) of the studied mice respect to the work of Tare et al. No relevant deviations were evidenced in flight calvaria of both Wt and PTN-Tg mice when compared with ground and vivarium calvaria controls. This was in line with previous studies on astronauts where no microgravity induced deterioration of the non weight-bearing bones was observed [4]. On the contrary, occasionally it was observed an increase of the thickness of cranial bones consequent to the raised pressure in the skull for the redistribution of blood and fluid in a headward direction. At a cellular level, several authors have observed actin and microtubule cytoskeletal modifications in cells cultured in the space environment [50]–[54]. Previous data show that morphological changes in rat osteosarcoma cells (ROS17/2.8) [55], [56], mouse osteoblastic cells (MC3T3-E1) [57] and human osteoblast-like cells (MG63) [58] maintained in various microgravity-like conditions can occur. Interestingly, in our study histological analysis showed a morphological alteration of flight Wt mice osteocytes that was not detectable in PTN-Tg mice. How PTN transgene could prevent in the transgenic mice bone tissue cell morphology alteration observed in Wt bones is not known. A decreased bone mass could be the result of a decreased bone formation, an increased bone resorption, or both. In the attempt to clarify cell processes responsible for the reduction in bone mass observed in flight mice, we determined the expression level of some typical bone formation and resorption marker genes in the RNA extracted from bones of all groups of mice. The level of mRNA for all the bone specific markers tested, i.e. alkaline phosphatase (ALP), collagen type I (Coll I) and osteocalcin (OC), was decreased in Wt mice compared to the ground and vivarium controls. A decreased osteocalcin (OC) expression, but not alkaline phosphatase (ALP), was reported also by other authors in rats maintained in microgravity conditions for shorter periods [32]. This suggests a reduced osteoblast differentiation and/or activity in the animals housed in space. The difference existing between some of the literature data and our own observations related to the ALP expression could be explained by the different length of the animal permanence in space. In the flight PTN-Tg samples the reduction in the expression of collagen type I (Coll I) and OC was present, but it was much less than in the samples from Wt mice maintained in the same conditions. This supports the idea that in microgravity the PTN transgene could better sustain the replacement (proliferation, maturation and differentiation starting from osteogenic progenitors) of the functional osteoblasts lost as result of the microgravity effect. The study of the expression level of the typical osteoclast markers and, more important, the observed reduction of the osteoprotegerin (OPG) protein in the animal serum pointed to an enhancement of the bone resorption occurring in both Wt and PTN-Tg mice when maintained in microgravity conditions.

Hopefully, if the space agencies will take the “political” decision of a re-flight of the MDS payload, there would be the opportunity to fly additional animals to confirm and to expand some of the observations made during the first MDS mission: eventually this type of studies will contribute to the comprehension of the behavior of a mammalian skeleton during a long-term space mission and to the understanding of the role played by the PTN transgene in counteracting the microgravity induced bone loss.

## Materials and Methods

### Materials

TRIreagent ®, 10% formalin solution neutral buffered, acetone, absolute ethanol (Sigma-Aldrich s.r.l, Saint Louis, MO, USA), Phosphate-Buffered Saline pH 7.4 (PBS) (Invitrogen) were used to obtain and to store samples from mice dissected tissues.

### Animals

The MDS experimentation was approved by the American Institutional Animal Care and Use Committee (IACUC) with protocol n° FLT-09-070(KSC) as well as by the Ethics Committee of the Animal Facility of the National Institute for Cancer Research (IST, Genova, Italy) and by the Public Veterinary Health Department of the Italian Ministry of Health with protocol n° 4347-09/03/2009-DGSA.P. and performed in accordance with the principles expressed in the “Guide for the care and the use of laboratory animals” (Office of Science and Health Reports of the USA National Institute of Health, Bethesda, USA). Wild-type C57BLJ10 male mice were purchased from Jackson Laboratories and delivered to Charles River Laboratories and then to NASA Kennedy Space Center (KSC) Science Lab Specific Pathogen Free (SPF) animal facility. The mice that were not utilized for the MDS flight experiment were delivered to the animal facility of National Institute for Cancer Research (IST) and kept in SPF Individually Ventilated Cages (IVC) conditions until the ground and vivarium control experiments started. The originally published BDF PTN-Tg mice [59] were backcrossed in C57Bl/J10 for their better adaptability to MDS module in the animal facility of IST, Genoa (Italy). PTN-Tg male mice were sent from the animal facility of IST to Charles River Laboratories for rederivation, breeding and husbandry activities. Charles River Laboratories sent mice that participated to the MDS experimentation to the NASA KSC Science Lab SPF animal facility whereas the remaining animals were delivered to the animal facility of IST to be housed in the same conditions of the wild-type mice. Under vivarium conditions, mice were kept in rooms characterized by 20–24°C temperature, 40–60% relative humidity and 12 hours of light/dark cycle. Food (Mucedola srl, Milan, Italy) and water were provided *ad libitum* to the vivarium experiment mice. For the animals housed in the MDS modules during flight and ground control experiment only water was provided *ad libitum* while 5 g/die food was automatically provided by the MDS module. IVC system cages had a size of 300 mm×160 mm×140 mm while cages of 330 mm×150 mm×120 mm were used for the vivarium control experiment. Additional information are given in the companion article of this collection “The Mice Drawer System (MDS) Experiment and the Space Endurance Record-Breaking Mice” by Cancedda et al.

### Animal habitat

The MDS model has been designed by Thales Alenia (Milano, Italy) and consists of one external container [5] with dimensions of 421 × 480 × 516 mm in which various subsystems are integrated. All the subsystems are studied in order to support animal survival for a period much longer than the scheduled experimental time of 100 days. The subsystems integrated in the MDS module are principally the following: Mice Chamber (MC), Liquid Handling Subsystem (LHS), Food Delivery Subsystem (FDS), Air Conditioning Subsystem (ACS), Illumination Subsystem (ILS), Observation Subsystem (OSS) [60]. Additional information are given in the companion article of this collection “The Mice Drawer System (MDS) Experiment and the Space Endurance Record-Breaking Mice” by Cancedda et al.

### Blood and serum analysis

Serum was obtained from blood upon drawing and stored at −80°C. Serum cytokines analysis was performed using Luminex^®^ technology instruments and Milliplex reagents (Millipore, Billerica, MA) following the manufacturer’s instructions. Serum was obtained centrifuging whole blood for 10′ at 3,000 rpm. Supernatant was stored at −80°C until analysis of osteoprotegerin (OPG) levels started. Due to the reduced number and amount of the samples, statistical analysis could not be performed and only standard deviations were calculated in internal repeats of the experiment.

### Synchrotron radiation X-Ray computed microtomography analysis

Synchrotron Radiation X-Ray *μ*CT imaging was performed at the SYRMEP Beamline of the ELETTRA Synchrotron Radiation Facility (Trieste, Italy). The 1200 radiographic projections were acquired with a beam energy of 19 keV over 180° with a resulting voxel size of 9 µm. The tomographic reconstruction was performed by means of the common filtered back-projection method. Investigations were focused on the lower third of the left femurs from the patella towards the shaft of the femur. The analysis of the trabecular structure was limited to a restricted sub-volume, which corresponded to the maximum rectangular prism (about 5 mm high) registrable inside the inner cortical wall. In the case of cortical analysis, a 540 µm thick portion 4 mm far away from the patella was considered. In the lumbar spine district the analysis was focused onto the Vertebral Body in the VII Lumbar ring and the maximum rectangular prism (about 2 mm high) recordable inside the inner cortical wall was selected and subjected to quantitative analysis. For the analysis of the non weight-bearing bones (calvaria), a 460 µm thick portion was selected in the middle of the parietal bone left portion and in transverse direction from the sagittal suture to the border. The following quantitative descriptors for trabecular bone architecture were extracted by means of the commercial software VG Studio Max: total volume (TV – expressed in µm^3^), bone volume (BV – expressed in µm^3^), bone volume to total volume ratio (BV/TV – expressed as a percentage), bone surface to bone volume ratio (BS/BV – per millimeter), trabecular thickness (Tb.Th – expressed in micrometers), trabecular number (Tb.N – per millimeter), and trabecular separation (Tb.Sp – expressed in micrometers). Skeleton analysis was also applied in order to derive a descriptor for the interconnectivity. By scanning the skeleton it is possible to extract the number of nodes and branches and, provided that the trabecular network is a connected structure with no closed void cavities, an indicator of the connectedness of the 3D complex pore space is the Euler number *χV = n−b* where *n* is the number of nodes and *b* the number of branches. It provides a measure of connectivity indicating the number of redundant connections (up to several hundreds) per mm^3^: the breaking of a single connection (skeleton branch) will leave the network less connected increasing the value of χV, while the addition of a redundant connection will decrease it. In order to normalize the Euler number with respect to the size of the considered volume V, the parameter connectivity density *β* computed as *β = (1−χV)/V* is commonly adopted [61]. The connectivity density gives higher values for better-connected structures and lower values for poorly connected structures. Structural anisotropy and connectivity analysis were performed by means of the Pore3D software library [62].

### Histology

After mice sacrifice, femurs from Wt and PTN-Tg mice were collected. Samples were fixed in PBS buffered 10% formalin fixative overnight, then right femurs were washed 3 times 5 minutes/wash with PBS and stored in 70% Ethanol at 4°C, whereas left corresponding bones were washed 3 times 5 minutes/wash directly with acetone before storage in acetone at 4°C. Due to the limited number of flight and ground bones available, the fixed samples were analyzed by *μ*CT prior their resin embedding. To obtain the resin embedded samples, bone samples were infiltrated with the light-curing resin Technovit 7200 VLC (Kulzer, Wehrheim, Germany) for 21 days under vacuum, changing the resin every 7 days. Samples were polymerized by the EXAKT 520 polymerization system (EXAKT Technologies, Oklahoma City). Curing was performed at 450 nm light with the temperature of the specimens never exceeding 40°C. The specimens were then prepared to be cut, according to the precision paralleling-guide procedure protocol, using the precision presses Exakt 401 and 402 Vacuum Adhesive Press (EXAKT Technologies, Oklahoma City). Sections were then cut using the EXAKT 310 CP cutting unit (EXAKT Technologies, Oklahoma City). The sections obtained were approximately 200 µm in thickness. Sections were then grinded up to 20–30 µm using the EXAKT 400 CS micro grinding unit (EXAKT Technologies, Oklahoma City). Sections were stained with Stevenel’s/Van Gieson stain. Images were taken using Axiovert 200M microscope (Zeiss, Germany). To determine osteocyte dimensions, we measured the width (minor diameter) of cells in femoral cortical bone using ImageJ software (http://rsbweb.nih.gov/ij/download.html). For each bone sample three sections were chosen and twenty fields/sections were analyzed. Width values were expressed in micrometers. Statistical analysis was performed by calculation of the *p* value using Graphpad software (http://www.graphpad.com/quickcalcs/ttest1.cfm).

### RNA extraction

Total RNA was extracted from tibia and humerus flushed bone marrow and separately from the remaining tibia and humerus bone tissue from Wt2, PTN-Tg1 and PTN-Tg2 mice using TRIreagent (Sigma Aldrich) following the manufacturer’s instructions. To extract RNA from bone as well as from the bone marrow, samples were mechanically disrupted with a homogenizer (UltraTurrax® homogenizer; IKA®-Werke GmbH & Co, Staufen, Germany).

### Reverse-transcriptase and real time polymerase chain reaction

cDNA was obtained using Omniscript RT Kit (Qiagen) following the manufacturer’s instructions. The gene expression analyses were performed by real time quantitative PCR using PE ABI PRISM 7700 sequence detection system (Perkin Elmer, MA) and SYBR Green (Applied biosystems, CA). Forward and reverse primer sequence were: GAPDH forward 5′-GATGCCTGCTTCACCACCTT-3′, reverse 5′-GATGCCTGCTTCACCACCTT-3′; CTK forward 5′-CACTCTCCAGAGTCGAGTC-3′, reverse 5′-GTGGTCTGCAGCATCGCT-3′; TRAP forward 5′-GACAAGAGGTTCCAGGAGACC-3′, reverse 5′-GGGCTGGGGAAGTTCCAG-3′; Coll I forward 5′-CTTGGTGGTTTTGTATTCGATGAC-3′, reverse 5′-GCGAAGGCAACAGTCGCT-3′; ALP forward 5′-GGACATCGCATATCAGCTAAT-3′, reverse 5′-GTATTCCACATCAGTTCTGTTC-3′; RankL forward 5′-GTGGTCTGCAGCATCGCT-3′, reverse 5′-CACTCTCCAGAGTCGAGTC-3′; OC forward 5′-CAGCGGCCCTGAGTCTGA-3′, reverse 5′-CCTCCTGCTTGGACATGAA-3′. The expression of GAPDH was examined as endogenous control. Relative transcripts levels were calculated from relative standard curve constructed from stock cDNA dilutions and divided by the target quantity of the calibrator following manufacturer’s instructions. Standard deviation values were derived from experimental internal repeats of the gene expression analysis.

## Supporting Information

Figure S1 **Histology on femurs.** Stevenel’s/Van Gieson staining was performed on the diaphysial region of the same *μ*CT analyzed femurs. Flight Wt2 (A) ground Wt2 (B) flight PTN-Tg2 (C) and ground PTN-Tg2 (D) cortical bone, magnification 20x. Osteocytes are visible as white spaces adjacent to “O”. P = periosteum, O = osteocytes, Bm = bone marrow.
(TIF)

Table S1 Morphometric parameters in Wt mice femur samples.
(DOC)

Table S2 Morphometric parameters in PTN-Tg mice femur samples.
(DOC)

Table S3 Morphometric parameters in Wt mice lumbar spine samples.
(DOC)

Table S4 Morphometric parameters in PTN-Tg mice lumbar spine samples.
(DOC)

Table S5 Acronyms. Acronyms reported and their meaning.
(DOC)
