# Supplementary figures and images for: Bone Turnover in Wild Type and Pleiotrophin-Transgenic Mice Housed for Three Months in the International Space Station (ISS)
Source: PLoS One. 2012 Mar 15;7(3):e33179. doi: 10.1371/journal.pone.0033179 (PMC3305296; doi:10.1371/journal.pone.0033179)

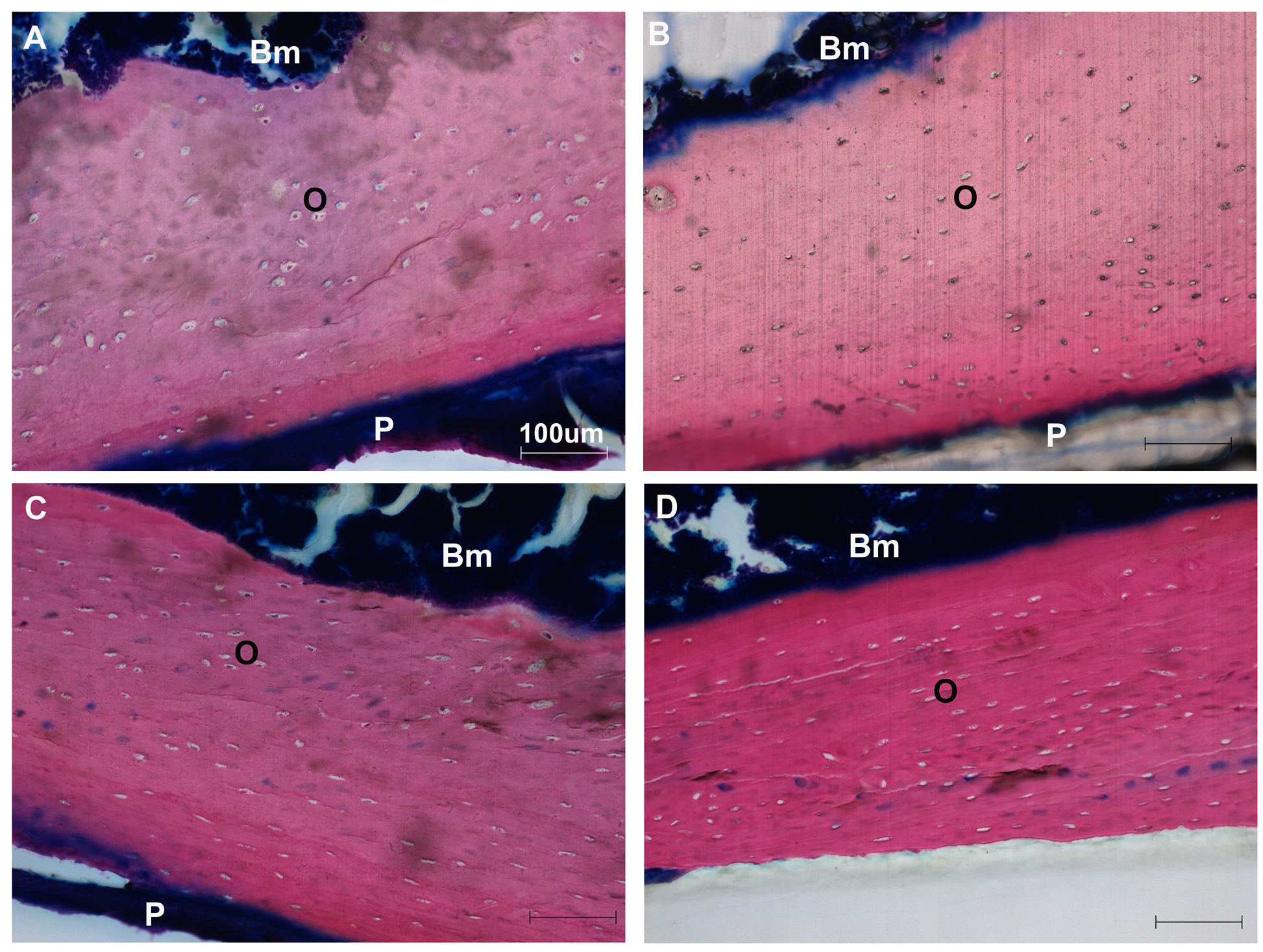

Supplement: Figure S1 — Histology on femurs. Stevenel’s/Van Gieson staining was performed on the diaphysial region of the same μCT analyzed femurs. Flight Wt2 (A) ground Wt2 (B) flight PTN-Tg2 (C) and ground PTN-Tg2 (D) cortical bone, magnification 20x. Osteocytes are visible as white spaces adjacent to “O”. P = periosteum, O = osteocytes, Bm = bone marrow. (TIF) [file pone.0033179.s001.tif]
