# Supplementary material for: Bone Turnover in Wild Type and Pleiotrophin-Transgenic Mice Housed for Three Months in the International Space Station (ISS)
Source: PLoS One. 2012 Mar 15;7(3):e33179. doi: 10.1371/journal.pone.0033179 (PMC3305296; doi:10.1371/journal.pone.0033179)
Supplement: Table S1 — Morphometric parameters in Wt mice femur samples. (DOC) [file pone.0033179.s002.doc]

|  | **VIVARIUM** | | **GROUND** | | | **FLIGHT** | | |
| --- | --- | --- | --- | --- | --- | --- | --- | --- |
|  | **Wt** | **Std.Dev.** | **Wt1** | **Wt2** | **Wt3** | **Wt1** | **Wt2** | **Wt3** |
| **days in MDS** | **0** |  | **44** | **91** | **16** | **44** | **91** | **16** |
| TV [µm^3^]: | 2.26E+09 | 0.02E+09 | 2.34E+09 | 3.11E+09 | 1.44E+09 | 2.90E+09 | 1.79E+09 | 3.31E+09 |
| BV [µm^3^]: | 7.46E+07 | 1.72E+07 | 7.49E+07 | 9.34E+07 | 6.61E+07 | 1.16E+07 | 0.89E+07 | 4.63E+07 |
| BS/BV [µm^-1^] | 0.102 | 0.005 | 0.099 | 0.114 | 0.098 | 0.087 | 0.128 | 0.120 |
| BV/TV [%] | 3.3 | 0.8 | 3.2 | 3.0 | 4.6 | 0.4 | 0.5 | 1.4 |
| Tb.Th [µm] | 20 | 1 | 20 | 18 | 21 | 23 | 16 | 17 |
| Tb.N [mm ^-1^] | 1.732 | 0.379 | 1.623 | 1.773 | 2.327 | 0.185 | 0.296 | 0.875 |
| Tb.Sp [µm] | 595 | 123 | 616 | 564 | 430 | 5411 | 3378 | 1142 |

**Table S1. Morphometric parameters in Wt mice femur samples.**

Acronyms reported in Table S1 are explained in Table S5.
