# Supplementary material for: Bone Turnover in Wild Type and Pleiotrophin-Transgenic Mice Housed for Three Months in the International Space Station (ISS)
Source: PLoS One. 2012 Mar 15;7(3):e33179. doi: 10.1371/journal.pone.0033179 (PMC3305296; doi:10.1371/journal.pone.0033179)
Supplement: Table S2 — Morphometric parameters in PTN-Tg mice femur samples. (DOC) [file pone.0033179.s003.doc]

|  | **VIVARIUM** | | **GROUND** | | | **FLIGHT** | | |
| --- | --- | --- | --- | --- | --- | --- | --- | --- |
|  | **PTN-Tg** | **Std.Dev.** | **PTN-Tg1** | **PTN-Tg2** | **PTN-Tg3** | **PTN-Tg1** | **PTN-Tg2** | **PTN-Tg3** |
| **days in MDS** | **-** |  | **91** | **91** | **24** | **91** | **91** | **24** |
| TV [µm^3^]: | 1.87E+09 | 0.29E+09 | 2.80E+09 | 2.29E+09 | 2.28E+09 | 2.07E+09 | 2.69E+09 | 2.26E+09 |
| BV [µm^3^]: | 2.85E+07 | 0.21E+07 | 6.71E+07 | 2.06E+07 | 4.56E+07 | 1.86E+07 | 1.08E+07 | 2.93E+07 |
| BS/BV [µm^-1^] | 0.117 | 0.003 | 0.116 | 0.110 | 0.094 | 0.114 | 0.106 | 0.111 |
| BV/TV [%] | 1.6 | 0.4 | 2.4 | 0.9 | 2.0 | 0.9 | 0.4 | 1.3 |
| Tb.Th [µm] | 17 | 0 | 17 | 18 | 21 | 18 | 19 | 18 |
| Tb.N [mm ^-1^] | 0.911 | 0.237 | 1.410 | 0.490 | 0.967 | 0.524 | 0.195 | 0.727 |
| Tb.Sp [µm] | 1136 | 295 | 709 | 2040 | 1034 | 1908 | 5141 | 1376 |

**Table S2. Morphometric parameters in PTN-Tg mice femur samples.**

Acronyms reported in Table S2 are explained in Table S5.
