# Supplementary material for: Bone Turnover in Wild Type and Pleiotrophin-Transgenic Mice Housed for Three Months in the International Space Station (ISS)
Source: PLoS One. 2012 Mar 15;7(3):e33179. doi: 10.1371/journal.pone.0033179 (PMC3305296; doi:10.1371/journal.pone.0033179)
Supplement: Table S3 — Morphometric parameters in Wt mice lumbar spine samples. (DOC) [file pone.0033179.s004.doc]

|  | **VIVARIUM** | | **GROUND** | | | **FLIGHT** | | |
| --- | --- | --- | --- | --- | --- | --- | --- | --- |
|  | **Wt** | **Std.Dev.** | **Wt1** | **Wt2** | **Wt3** | **Wt1** | **Wt2** | **Wt3** |
| **days in MDS** | **-** |  | **44** | **91** | **16** | **44** | **91** | **16** |
| TV [µm^3^]: | 1.04E+09 | 0.36E+09 | 1.47E+09 | 1.41E+09 | 0.27E+09 | 0.57E+09 | 0.728E+09 | 0.42E+09 |
| BV [µm^3^]: | 21.90E+07 | 5.16E+07 | 28.80E+07 | 28.0E+07 | 4.48E+07 | 6.84E+07 | 8.45E+07 | 5.27E+07 |
| BS/BV [µm^-1^] | 0.060 | 0.006 | 0.068 | 0.063 | 0.072 | 0.069 | 0.075 | 0.076 |
| BV/TV [%] | 22.0 | 4.1 | 19.6 | 19.8 | 16.5 | 11.9 | 11.6 | 12.5 |
| Tb.Th [µm] | 34 | 3 | 30 | 32 | 28 | 29 | 27 | 26 |
| Tb.N [mm ^-1^] | 8.382 | 1.440 | 8.238 | 7.784 | 7.091 | 4.651 | 4.894 | 5.397 |
| Tb.Sp [µm] | 121 | 19 | 121 | 128 | 141 | 215 | 204 | 185 |

**Table S3. Morphometric parameters in Wt mice lumbar spine samples.**

Acronyms reported in Table S3 are explained in Table S5.
