# Supplementary material for: Bone Turnover in Wild Type and Pleiotrophin-Transgenic Mice Housed for Three Months in the International Space Station (ISS)
Source: PLoS One. 2012 Mar 15;7(3):e33179. doi: 10.1371/journal.pone.0033179 (PMC3305296; doi:10.1371/journal.pone.0033179)
Supplement: Table S4 — Morphometric parameters in PTN-Tg mice lumbar spine samples. (DOC) [file pone.0033179.s005.doc]

|  | **VIVARIUM** | | **GROUND** | | | **FLIGHT** | | |
| --- | --- | --- | --- | --- | --- | --- | --- | --- |
|  | **PTN-Tg** | **Std.Dev.** | **PTN-Tg1** | **PTN-Tg2** | **PTN-Tg3** | **PTN-Tg1** | **PTN-Tg2** | **PTN-Tg3** |
| **days in MDS** | **-** |  | **91** | **91** | **24** | **91** | **91** | **24** |
| TV [µm^3^]: | 1.18E+09 | 0.15E+09 | 1.01E+09 | 0.62E+09 | 1.16E+09 | 1.24E+09 | 0.83E+09 | 1.16E+09 |
| BV [µm^3^]: | 20.20E+07 | 2.59E+07 | 19.30E+07 | 9.05E+07 | 24.90E+07 | 21.10E+07 | 11.80E+07 | 16.80E+07 |
| BS/BV [µm^-1^] | 0.065 | 0.005 | 0.063 | 0.066 | 0.062 | 0.065 | 0.060 | 0.075 |
| BV/TV [%] | 17.2 | 2.1 | 19.2 | 14.6 | 21.5 | 17.0 | 14.1 | 14.5 |
| Tb.Th [µm] | 31 | 3 | 32 | 30 | 32 | 31 | 33 | 27 |
| Tb.N [mm ^-1^] | 6.624 | 0.485 | 7.502 | 5.689 | 8.558 | 6.720 | 4.908 | 6.325 |
| Tb.Sp [µm] | 152 | 11 | 133 | 176 | 117 | 149 | 204 | 158 |

**Table S4. Morphometric parameters in PTN-Tg mice lumbar spine samples.**

Acronyms reported in Table S4 are explained in Table S5.
