# Supplementary material for: Bone Turnover in Wild Type and Pleiotrophin-Transgenic Mice Housed for Three Months in the International Space Station (ISS)
Source: PLoS One. 2012 Mar 15;7(3):e33179. doi: 10.1371/journal.pone.0033179 (PMC3305296; doi:10.1371/journal.pone.0033179)
Supplement: Table S5 — Acronyms. Acronyms reported and their meaning. (DOC) [file pone.0033179.s006.doc]

**Table S5: Acronyms.** Acronyms reported and their meaning.

| **Acronym** | **Acronym meaning** |
| --- | --- |
| ACS | Air Conditioning Subsystem |
| ALP | Alkaline phosphatase |
| ASI | Italian Space Agency |
| BS | Bone surface |
| BS/BV | bone surface to bone volume ratio |
| BV | Bone volume |
| BV/TV | total volume ratio |
| Coll I | Collagen type I |
| CRL | Charles River Laboratories |
| CTK | Cathepsin K |
| DXA | Dual-emission X-ray Absorptiometry |
| FDS | Food Delivery Subsystem |
| GAPDH | [Glyceraldehyde 3-phosphate dehydrogenase](http://en.wikipedia.org/wiki/Glyceraldehyde_3-phosphate_dehydrogenase) |
| IACUC | American [Institutional Animal Care and Use Committee](http://en.wikipedia.org/wiki/Institutional_Animal_Care_and_Use_Committee) |
| ILS | Illumination Subsystem |
| ISS | International Space Station |
| IVC | Individual Ventilated Cages |
| IVC | Individually Ventilated Cages |
| JSA | Japanese Space Agency |
| KSC | Kennedy Space Center |
| LED | Light-Emitting Diode |
| LHS | Liquid Handling Subsystem |
| MC | Mice Chamber |
| MDS | Mice Drawer System |
| NASA | National Aeronautics and Space Administration |
| OC | Osteocalcin |
| OPG | Osteoprotegerin |
| OSS | Observation Subsystem |
| PI | Principal Investigator |
| PTN | Pleiotrophin |
| PTN-Tg | Pleiotrophin transgenic |
| RankL | Rank ligand |
| SLSL | Space Life Science Laboratory |
| STS | Space Transportation System |
| Tb.N | Mean trabecular number |
| Tb.Sp | Mean trabecular separation |
| Tb.Th | Mean trabecular thickness |
| TRAP | tartrate resistant alkaline phosphatase |
| TSP | Tissue Sharing Program |
| TV | Total volume |
| ULF | Utilization and Logistic Flight |
| Wt | Wild type |
| *μ*CT | Computed microtomography |
